# Cancerous inhibitor of protein phosphatase 2A contributes to human papillomavirus oncoprotein E7-induced cell proliferation via E2F1

**DOI:** 10.18632/oncotarget.2867

**Published:** 2015-02-04

**Authors:** Weifang Zhang, Hanxiang Chen, Yan Chen, Juan Liu, Xiao Wang, Xiuping Yu, Jason J. Chen, Weiming Zhao

**Affiliations:** ^1^ Institute of Pathogenic Biology, Shandong University School of Medicine, Jinan, Shandong, China; ^2^ Institute of Pathophysiology, Shandong University School of Medicine, Jinan, Shandong, China; ^3^ Department of Medicine, University of Massachusetts Medical School, Worcester, MA, USA; ^4^ Cancer Research Center, Shandong University School of Medicine, Jinan, Shandong, China

**Keywords:** CIP2A, HPV, E7, G1 arrest, E2F1

## Abstract

Cancerous inhibitor of protein phosphatase 2A (CIP2A) is a recently identified oncoprotein that is overexpressed in many human malignant tumors including cervical cancer. Human papillomavirus (HPV) oncoprotein E7 is the key transformation factor in cervical cancer. Our previous data showed a positive association of CIP2A and HPV-16E7 protein levels; however, how CIP2A is regulated by HPV-E7 and the roles of CIP2A in HPV-E7-mediated cell proliferation are unknown. In this study, we demonstrated that HPV-16E7 protein significantly upregulating CIP2A mRNA and protein expression depended on retinoblastoma protein pRb rather than p130. CIP2A siRNA knockdown in HPV-E7-expressing cells inhibited cell proliferation, DNA synthesis and G1/S cell cycle progression. CIP2A siRNA decreased the protein levels of cyclin-dependent kinase 1 (Cdk1), Cdk2 and their partner cyclin A2, with no change in levels of Cdk4, Cdk6 and their partner cyclin D1. The downregulation of Cdk1 and Cdk2 was independent of c-Myc; instead, E2F1 was the main target of CIP2A in this process, as overexpression of E2F1 rescued the inhibitory effects of CIP2A siRNA knockdown on cell proliferation and G1 arrest of HPV-E7-expressing cells. Our studies reveal a novel function of CIP2A in HPV-16E7-mediated cell proliferation.

## INTRODUCTION

Cervical cancer is one of the most common malignancies in women worldwide; the morbidity is second only to breast cancer [1]. Human papillomavirus (HPV) is present in more than 99% of cervical cancers [2] and high-risk HPV is the key carcinogenic factor in carcinoma of cervical epithelial cells [3, 4]. The HPV genome, a double-stranded DNA, contains oncogenes E6 and E7, which play a key role in carcinogenesis. E6 and E7 can bind and inactivate tumor suppressors p53 and the retinoblastoma protein family (pRb, p130 and p107), respectively, thus modulating key cellular processes such as proliferation and transformation [5, 6]. Degradation of pRb results in its dissociation from members of the E2F family of transcription factors. Free E2Fs activate the transcription of genes required for DNA synthesis and promote cells entering the S phase [7].

Cancerous inhibitor of protein phosphatase 2A (PP2A; CIP2A) was first identified as a novel endogenous interacting partner of PP2A [8], which is a serine/threonine phophatase and may function as a tumor suppressor [9, 10]. Later CIP2A was found to be overexpressed in many human malignancies including gastric, bladder, ovarian, tongue, hepatocellular, and colon cancer as well as non-small cell lung carcinoma and chronic myelogenous leukemia (reviewed in [11]). Moreover, CIP2A overexpression has been associated with tumor grade and poor prognosis in many cancers [11]. CIP2A promotes cancer cell proliferation and renders cancer cells resistant to therapy-induced apoptosis or senescence [11]. The expression of CIP2A may be used as a biomarker for screening, diagnosis and prognosis in many cancers [12]. Inhibition of PP2A, which dephosphorylates c-Myc at serine 62 (S62) and leads to proteolytic degradation of c-Myc, is one mechanism of CIP2A executing its oncogenic function [8]. CIP2A stabilizes c-Myc by inhibiting PP2A, but c-Myc stimulates CIP2A mRNA and protein expression in gastric cancer [13]. Despite the oncogenic role of CIP2A in human malignancies, how it regulates cell proliferation and the cell cycle are still unknown.

Cell cycle progression is regulated by cyclins and cyclin-dependent kinases (Cdks) at several checkpoints [14]. The G1 checkpoint is the master checkpoint to determine whether cells enter the S phase and proliferate. HPV-16E7 abrogates the G1 cell-cycle checkpoint by degrading pRb (reviewed in [15]), but whether CIP2A is involved in this process is unclear.

Recently, we showed that CIP2A was overexpressed in cervical cancer, and its expression was associated with tumor progression [16]. As well, CIP2A protein level was found positively associated with HPV-16E7 level in cervical cancer tissue [16]. However, how CIP2A is regulated by E7 and the role of CIP2A in E7-expressing cells and in the pathogenesis of cervical cancer are unknown. In the present study, we analyzed the expression of CIP2A protein in E7-expressing primary human keratinocytes (PHKs) and investigated the function of CIP2A in cell proliferation and the cell cycle in HPV-E7-expressing cells.

## RESULTS

### CIP2A upregulation by high-risk HPV-E7 depends on pRb but not p130

**Figure 1:**
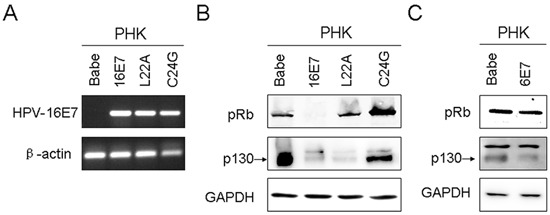
Expression of human papillomavirus (HPV)-16E7, 6E7 and 16E7 mutants in primary human keratinocytes (PHKs) **(A)** RT-PCR of HPV-16E7 in PHKs expressing 16E7 and 16E7 mutants L22A and C24G. β-actin was used as a control. **(B)** Western blot analysis of pRb and p130 protein in PHKs expressing 16E7 and 16E7 mutants L22A and C24G. GAPDH was a loading control. **(C)** Western blot analysis of pRb and p130 protein in PHKs expressing 6E7. Babe, vector control.

We previously showed a positive association of CIP2A and HPV-16E7 immunoreactivity, and HPV-16E7 depletion in cervical cancer SiHa cells significantly reduced CIP2A expression [16]. To explore whether CIP2A is directly modulated by HPV-16E7, we established PHKs expressing HPV-16E7 by retrovirus-mediated successive infection; the expression was confirmed by RT-PCR (Fig. 1A). Pocket protein pRb and p130 levels were both reduced in PHKs expressing HPV-16E7, so HPV-16E7 was functionally active (Fig. 1B).

**Figure 2:**
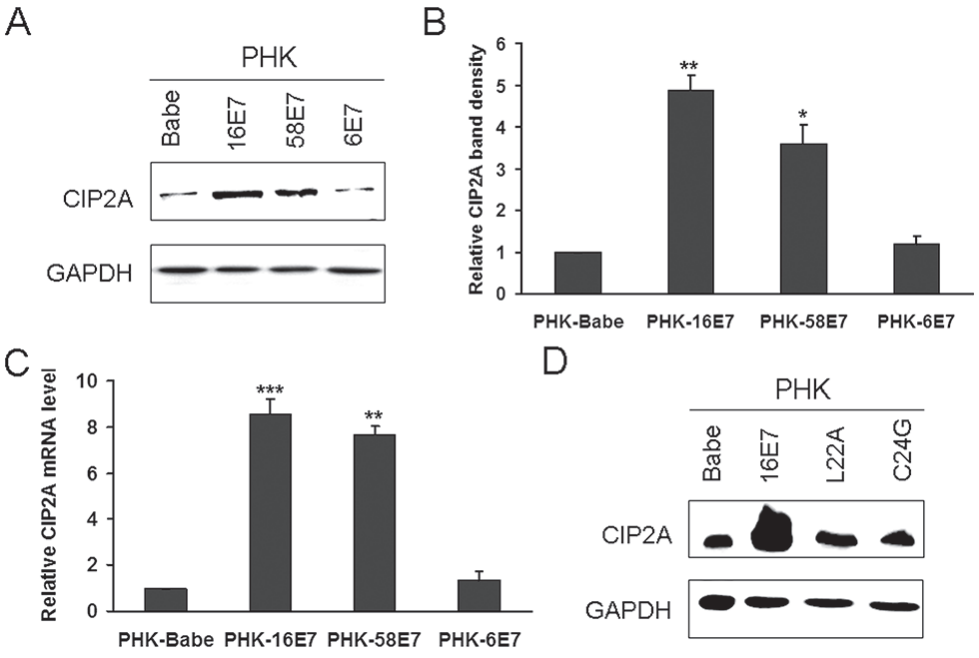
HPV-16E7 and -58E7 upregulated CIP2A mRNA and protein levels in PHKs **(A)** Western blot analysis of CIP2A protein level in PHKs expressing HPV-16E7, -58E7, -6E7; and **(B)** Quantification. **(C)** qRT-PCR analysis of CIP2A mRNA level in PHKs expressing HPV-16E7, -58E7, -6E7. **(D)** Western blot analysis of CIP2A protein level in PHKs expressing HPV-16E7 and 16E7 mutants L22A and C24G. Babe, vector control. *, *P* < 0.05; **, *P* < 0.01; and ***, *P* < 0.001.

Next we detected CIP2A protein level in HPV-16E7-expressing PHKs. HPV-16E7 significantly upregulated CIP2A protein level (about 4.8-fold; *P* < 0.01) as compared with the control (Fig. 2A and 2B). CIP2A mRNA level was increased about 8.6-fold (*P* < 0.001) in HPV-16E7-expressing PHKs (Fig. 2C). To explore whether the upregulation of CIP2A by HPV-16E7 was type-specific, we examined its expression in cells expressing HPV-58E7, the third most common HPV type associated with cervical cancer in Eastern Asia [17]. CIP2A protein level was upregulated about 3.6-fold by HPV-58E7 (Fig. 2A and 2B), and CIP2A mRNA level was upregulated about 7.5-fold (Fig. 2C), but with lower efficiency as compared with HPV-16E7. To further explore whether the effect of E7 on CIP2A expression is unique to high-risk E7, we established low-risk HPV type-HPV 6E7-expressing PHKs. Consistent to what was reported before [18], HPV 6E7 did not target pRb for degradation, instead, 6E7 degraded p130 efficiently (Fig. 1C). As expected, there are no significant differences in CIP2A protein and mRNA expressions between 6E7-expressing PHKs and control cells (Fig. 2A, 2B and 2C). These data indicate that up-regulating CIP2A is high-risk HPV type specific.

E7 is the major transforming protein expressed by HPV and its transformation activity mainly relies on its LXCXE motif (residues 22 to 26), which is critical for binding pocket proteins. Therefore, mutation of the LXCXE motif of HPV-16E7 abrogates high-affinity binding to pocket proteins and transformation activity [19]. To determine whether the LXCXE motif is important in modulating CIP2A expression, we used the E7 mutant L22A, which is defective in binding to pRb but can bind p130, and the mutant C24G, which is defective in binding both pRb and p130. The expression of L22A and C24G was confirmed by RT-PCR (Fig. 1A), Western blot analysis showed that these two mutants were active (Fig. 1B). Not surprisingly, the E7 mutants L22A and C24G, which can not degrade pRb, did not increase CIP2A protein level (Fig. 2D). Although the E7 mutant L22A degraded p130 efficiently (Fig. 1B), the protein level of CIP2A in PHK-L22A remained nearly the same as in control cells (Fig. 2D). Thus, HPV-16E7 upregulating CIP2A expression in PHKs depended on pRb but not p130 degradation.

### CIP2A plays a role in proliferation and S-phase entry in E7-expressing cells

**Figure 3:**
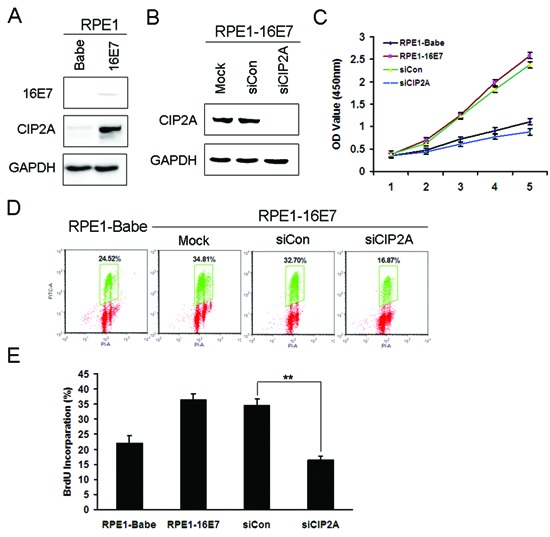
Knockdown of CIP2A inhibited cell proliferation and DNA synthesis of HPV-16E7-expressing cells **(A)** Western blot analysis of protein level of 16E7 and CIP2A in RPE1-16E7 cells and **(B)** with CIP2A siRNA for 48 hr. **(C)** CCK8 assay of cell proliferation of RPE1-16E7 cells with CIP2A siRNA. **(D)** Flow cytometry of cells with CIP2A siRNA and labeled with BrdU for 2 hr, then stained with PI and BrdU; and **(E)**, Quantification. Babe, vector control. **, *P* < 0.01.

CIP2A was reported to promote cell growth [11]. To elucidate whether CIP2A contributes to the proliferation of E7-expressing cells, we trasnfected CIP2A-specific small interfering RNA (siRNA) which was proved to be specific and effective to knock down CIP2A expression [16]. As PHKs are not only difficult to transfect, but also arrest at the G1 phase upon transfection, we used human retinal pigment epithelium cells (RPE1) expressing HPV-16E7 for siRNA knockdown experiments. The expression of HPV-16E7 in these cells was detected previously [20]. Before we conducted the siRNA experiments, we confirmed the expression of 16E7 (Fig. 3A). HPV-16E7 increased CIP2A protein expression in RPE1 cells (Fig. 3A). CIP2A protein level was efficiently knocked down with siRNA (Fig. 3B). CIP2A knockdown severely impaired the growth of RPE1-16E7 cells within 5 days, and cell proliferation was significantly slower than the scrambled siRNA control cells, close to RPE1-Babe control cells (Fig. 3C).

To examine whether CIP2A knockdown in E7-expressing cells affected DNA replication, we measured bromodeoxyuridine (BrdU) incorporation. CIP2A knockdown significantly reduced the number of BrdU-positive cells (16.87% vs 32.70%) (Fig. 3D), with a significant difference compared with scrambled siRNA knockdown (*P* < 0.01) (Fig. 3E). Thus, CIP2A knockdown in HPV-16E7-expressing cells impaired DNA synthesis and affected cells entering the S phase.

### CIP2A siRNA knockdown in E7-expressing cells causes G1 arrest

**Figure 4:**
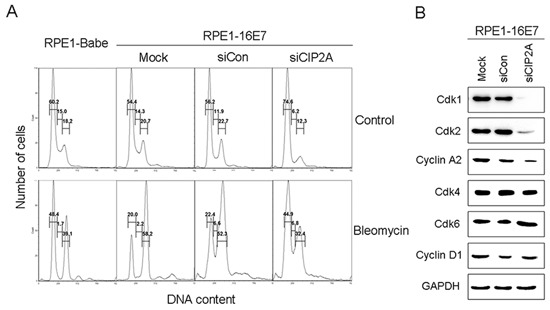
CIP2A siRNA knockdown caused G1 arrest and decreased Cdk1 and Cdk2 protein levels in E7-expressing cells **(A)** Flow cytometry of cells expressing 16E7 transfected with CIP2A siRNA for 48 hr, treated with DMSO control or bleomycin (10 μg/mL) for 24 hr, then stained with PI. G1, S and G2 phases are indicated. **(B)** Western blot analysis of Cdk1, Cdk2, Cyclin A2, Cdk4, Cdk6, Cyclin D1 protein levels in cells expressing HPV-16E7 transfected with CIP2A siRNA. Babe, vector control.

Because HPV-16E7 abrogates the G1 cell-cycle checkpoint (reviewed in [15]), we examined the ability of CIP2A to modulate the G1 checkpoint in E7-expressing cells by treating cells with bleomycin, a chemical agent capable of inducing both single- and double-stranded DNA breaks that lead to cell cycle arrest at G1 checkpoints [21]. CIP2A knockdown caused more cells to arrest in the G1 phase (74.6% vs 56.2%) and fewer cells (6.2% vs 11.9%) entering the S phase (Fig. 4A). Consistent with our previous data [20], with bleomycin treatment, about 48.4% of RPE1-Babe vector cells arrested in the G1 phase, as compared with 20.0% of RPE1-16E7-expressing cells, indicating that 16E7 bypassed bleomycin-induced G1 arrest. Importantly, CIP2A knockdown affected the ability of E7 to overcome G1 arrest by causing an increased proportion of cells arrested in the G1 phase (44.9% vs 22.4% for siRNA control), close to the proportion of RPE1-Babe vector cells.

Therefore, CIP2A knockdown in E7-expressing cells impaired G1/S cell cycle progression and attenuated the ability of HPV-16E7 to abrogate the DNA damage-induced G1 checkpoint.

### CIP2A siRNA knockdown downregulates cell cycle related proteins

Cell cycle progression is regulated by cyclins and Cdks at several checkpoints, with the G1 checkpoint the most important. Cdk2 is a major regulator in S phase entry, but its function can be compensated by Cdk1 in its absence (reviewed in [22]). To explore how CIP2A regulates G1 checkpoint, we examined the expression of genes involved in the G1 checkpoint, including all G1 Cdks – Cdk1, Cdk2, Cdk4, Cdk6 and their associated cyclin partners – cyclin D1 and A2. The steady-state levels of Cdk2 and cyclin A2 as well as the mitotic Cdk, Cdk1, were downregulated with CIP2A knockdown, with no significant effect of CIP2A knockdown on steady-state levels of Cdk4 (Fig. 4B). The level of Cdk6, which is structurally and functionally similar to Cdk4, was unchanged as well (Fig. 4B). Correspondingly, the level of the partner of Cdk4 and Cdk6, the main D type cyclin, cylin D1, remained almost the same in control and CIP2A knockdown cells (Fig. 4B). These results suggest a role of Cdk1 and Cdk2 in impairing G1/S transition with CIP2A knockdown in E7-expressing cells.

### Downregulation of Cdks by CIP2A siRNA knockdown in E7-expressing cells depends on ‘active E2Fs’ rather than c-Myc

**Figure 5:**
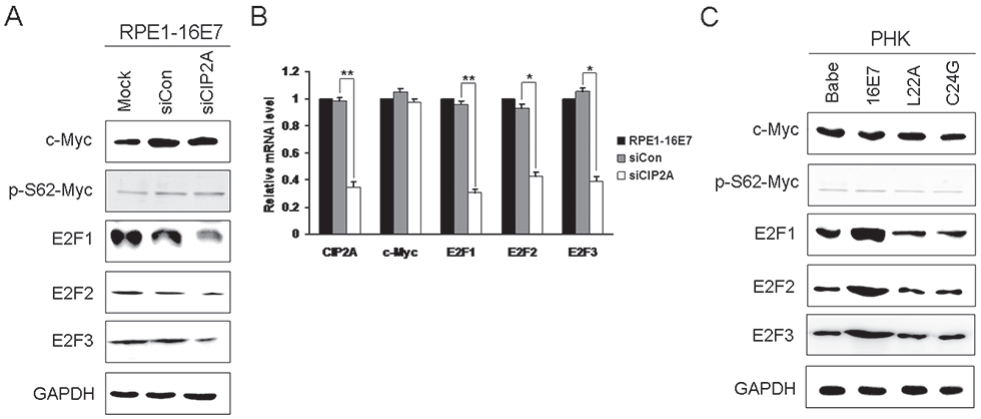
Active E2Fs expression positively associated with CIP2A expression in HPV-16E7-expressing cells **(A)** Western blot analysis of c-Myc, phosphor-S62-Myc, E2F1, E2F2 and E2F3 protein levels and **(B)** qRT-PCR analysis of CIP2A, c-Myc, E2F1, E2F2 and E2F3 mRNA levels in RPE1-16E7 cells with CIP2A siRNA. **(C)** Protein levels of c-Myc, phosphor-S62-Myc, E2F1, E2F2 and E2F3 in PHKs expressing 16E7 and E7 mutants L22A and C24G. Babe, vector control. *, *P* < 0.05; **, *P* < 0.01.

CIP2A stabilizes the c-Myc oncoprotein, an important transcription modulator regulating cell proliferation, differentiation, cell cycle, apoptosis and cellular senescence [23]. Because CIP2A can stabilize PP2A-associated c-Myc, we speculated that CIP2A-mediated cell-cycle regulation may depend on c-Myc. Unexpectedly, both the protein and mRNA levels of c-Myc remained the same with CIP2A knockdown in HPV-16E7 cells (Fig. 5A and 5B). The abundance of c-Myc protein is also affected by many kinases which phosphorylate different sites of c-Myc, inhibiting or promoting its degradation [24]. Since CIP2A is reported to stabilize c-Myc by preventing PP2A-mediated dephosphorylation on S62 of c-Myc, we further examined expression of phosphor-S62-Myc. However, the protein expression of phosphor-S62-Myc did not alter after CIP2A siRNA knockdown. Instead, inhibition of CIP2A expression resulted in robust reduction of both mRNA and protein levels of E2F1, E2F2 and E2F3, which are generally considered as ‘active E2Fs’ as their binding to promoters results in increased transcription [25] (Fig. 5A and 5B). Therefore, CIP2A knockdown downregulating late G1 Cdks and cyclins may depend on active E2Fs rather than c-Myc. To clarify whether CIP2A and active E2Fs or c-Myc expression was positively related, we examined their expressions in PHKs expressing 16E7 and E7 mutants. Similar to the CIP2A expression pattern, E7 but not E7 mutants greatly increased the expression of active E2Fs, while the expression of c-Myc and phosphor-S62-Myc was not altered by HPV E7 (Fig. 5C). Therefore, in E7-expressing cells, CIP2A and active E2Fs expression was positively related.

**Figure 6:**
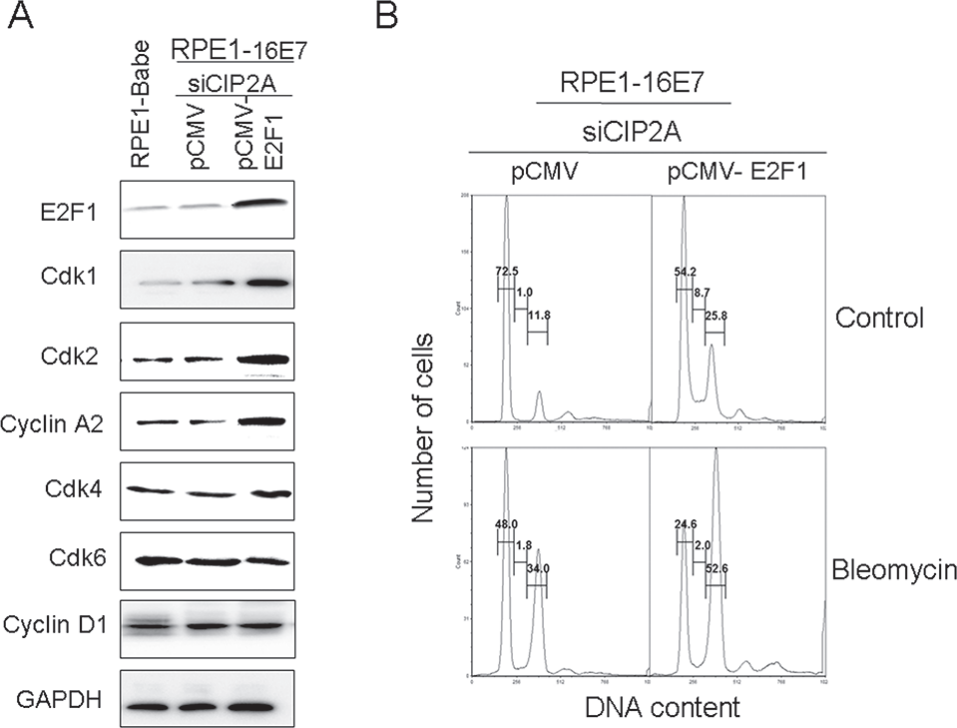
Overexpression of E2F1 overcame G1 arrest induced by CIP2A siRNA knockdown and upregulated expression of Cdk1 and Cdk2 protein in 16E7-expressing cells **(A)** Western blot analysis of E2F1, Cdk1, Cdk2, cyclin A2, Cdk4, Cdk6 and Cyclin D1 protein levels in RPE1–16E7 cells transfected with CIP2A siRNA, then transfected with pCMV or pCMV-E2F1 plasmid. **(B)** Flow cytometry of cells treated with/out bleomycin stained with PI. G1, S and G2 phase are indicated.

We wondered whether CIP2A regulates E2Fs activity during G1/S cell cycle progression. Considering the importance of their roles in cell proliferation, E2F1 was selected for the following experiments. To test whether CIP2A knockdown downregulated Cdks and caused G1 arrest by E2F1, we overexpressed E2F1 to determine whether it rescued the inhibitory effect of CIP2A knockdown. After transfection of E2F1 plasmid into E7 cells with CIP2A knockdown, the expression of E2F1 was first confirmed (Fig. 6A). As expected, the expression of Cdk1, Cdk2 and cyclin A2 increased significantly in E2F1-overexpressing cells, meanwhile, the expression of Cdk4, Cdk6 and cyclin D1 remained unchanged (Fig. 6A). With overexpression of E2F1, cells with CIP2A knockdown overcame the G1 arrest and fewer cells arrested in the G1 stage (54.2% vs 72.5%) and more cells entered the S phase (8.7% vs 1.0%) (Fig. 6B). With bleomycin treatment, E2F1 overexpression overcame the G1 arrest caused by CIP2A knockdown, for fewer cells arrested in the G1 stage (24.6% vs 48.0%) (Fig. 6B).

Thus, restoring E2F1 function in CIP2A-depleted cells upregulated Cdk1 and Cdk2 expression, and E2F1 overexpression rescued the inhibitory effect of CIP2A knockdown on G1 arrest in E7-expressing cells.

## DISCUSSION

Our previous study showed that CIP2A was overexpressed in cervical cancer and positively related with HPV-16E7 expression [16]. The data in the present study show that HPV-16E7 significantly upregulated CIP2A in primary PHKs. A recent study showed that E2F1 directly binds to the CIP2A promoter and regulates CIP2A transcription [26]. Because the HPV oncoprotein E7 inactivates the tumor suppressor pRb and releases E2F1 [27], CIP2A expression should be increased in E7-expressing cells. Accordingly, E7 mutants defective in the interaction between E7 and pRb did not show increased CIP2A protein level. Although as a member of the pRb family, p130 has overlapping functions with pRb, they function at different phases of the cell cycle. The expression of pRb is maintained throughout the whole cell cycle, whereas p130 mainly plays a role in maintaining a quiescent state [28]. Besides, they have distinct functions, for example, loss of Rb in the mouse leads to embryonic lethality [29], whereas loss of p130 has no discemable effect [30]. As well, mutation of pRb but not p130 occurs frequently in nearly all human cancers [31], which explains to a certain extent why degradation of pRb but not p130 is necessary in upregulating CIP2A level. However, the molecular basis of this regulation needs further study.

Our results revealed that CIP2A was associated with cell cycle regulation. In accordance with previous reports [32], CIP2A knockdown increased the proportion of ovarian cancer cells in the G1 phase and decreased that in the S phase, so CIP2A regulated cell cycle progression at the G1/S boundary. Recent knockout mouse models demonstrated that progression through the G1/S transition is associated with the activity of four cdks – Cdk1, Cdk2, Cdk4, and Cdk6 and their cyclin regulators [22, 33]. A recent study demonstrated that Cdk1 can bind cyclin E and substitute for Cdk2 during the G1/S phase transition [34]. In addition, mouse embryonic fibroblasts from Cdk2/Cdk3/Cdk4/Cdk6-knockout mice showed that Cdk1 binds all cyclins and phosphorylates pRb, releasing E2F and accelerating the cell cycle [35]. Thus, in the absence of these G1 Cdks, Cdk1, which normally functions at the G2 phase, can substitute for these Cdks and function in the G1/S phase transition. Our data showed that both Cdk2 and Cdk1 function in the G1/S transition and downregulated Cdk2 and Cdk1 contribute to G1 arrest caused by CIP2A siRNA knockdown.

c-Myc is an “immediate early gene” in the response to growth-stimulating signals and an important positive regulator of cell proliferation. Because CIP2A inhibits PP2A-mediated dephosphorylation of the c-Myc on S62, resulting stabilization of c-Myc [13], we speculated that CIP2A-mediated cell cycle regulation may depend on c-Myc. Unexpectedly, both the mRNA and protein levels of c-Myc remained unchanged with CIP2A knockdown. Besides, the expression of phosphor-S62-Myc did not change with CIP2A knockdown as well. Although *in vitro* experiments showed that HPV-16E7 interacts with and forms a specific complex with c-Myc and augments c-Myc transactivation activity [36], the regulation of c-Myc expression by HPV-E7 protein is complex. Introduction of the HPV-E7 oncogene has no consistent effect on c-Myc expression: the increased c-Myc protein level found in some studies may be a primary effect of E7 expression or an effect of E7-induced fast growth rate [37–39]. Our results showed no increase in c-Myc level in keratinocytes expressing E7 (Fig. 5C). Because c-Myc is an “immediate early gene” in the response to growth-stimulating signals, while we cultured the keratinocytes for several weeks, c-Myc may be differentially modulated during successive passaging of human keratinocytes expressing HPV-16E7, as observed previously [37]. In terms of c-Myc regulation by CIP2A, some laboratories have shown decreased c-Myc level in CIP2A-depleted cells [8, 13, 32, 40, 41]. These findings did not agree with our current observation that CIP2A knockdown did not affect c-Myc level; however, these previous reports examined c-Myc levels after CIP2A knockdown in cancer cells, whereas we observed c-Myc expression in HPV-16E7-expressing cells, with no increase in c-Myc expression. In addition, a previous report showed comparable CIP2A expression in S-phase synchronized and unsynchromized gastric cancer cells, so cell cycle activity may not be associated with c-Myc-mediated regulation of CIP2A expression in gastric cancer cells [13]. Therefore, CIP2A-dependent cell cycle regulation may be independent of c-Myc.

A recent study revealed that E2F1 activates CIP2A by binding the promoter of CIP2A, whereas CIP2A stabilizes E2F1 by preventing PP2A-mediated dephosphorylation of the E2F1 on Ser 364 [26]. We noted that both CIP2A and E2F1 were upregulated by HPV-E7, not the E7 mutant deficient in binding and degrading pRb. As well, the mRNA and protein levels of E2F1 decreased significantly with CIP2A knockdown in E7-expressing cells. These results demonstrate a positive association of CIP2A and E2F1 in E7-expressing cells and suggest that CIP2A regulating Cdks may depend on E2F1. E2F regulates cyclin A and Cdk1 transcription [42, 43] and E2F binding sites have been found in the upstream regions of the human cyclin A, Cdk1 and Cdk2 genes [44–46]. Because Cdk1, Cdk2 and cyclin A2 are key proteins in the G1/S transition, CIP2A knockdown may impede the G1/S progression by downregulating these proteins via E2F1 in E7-expressing cells. Although E2F1 expression was decreased with CIP2A knockdown, whether CIP2A binds to the promoter of E2F1 and transactivates it remains to be determined.

In summary, we demonstrate that CIP2A was upregulated by high-risk HPV-E7 depending on pRb but not p130 degradation. CIP2A is important for the G1/S cell cycle progression in HPV-E7-expressing cells. Moreover, CIP2A regulating Cdk1 and Cdk2 expression in E7-expressing cells depends on E2F1. These results suggest the specific functional roles of CIP2A in HPV-E7-mediated regulation of cell cycle progression and proliferation that may contribute to HPV-induced carcinogenesis. The discovery of CIP2A as a regulator in HPV-associated cancer is of clinical importance for cancer diagnosis and potential therapeutic targeting.

## MATERIALS AND METHODS

### Cell culture

Primary human keratinocytes (PHKs) were derived from neonatal human foreskin epithelium obtained from the University of Massachusetts Hospital as described [47]. PHKs were maintained on mitomycin C-treated J2-3T3 feeder cells in F-medium composed of 3 parts Ham's F12 medium, 1 part DMEM, and 5% fetal bovine serum (FBS; all Invitrogen) with all supplements described [48]. Human retinal pigment epithelial (RPE1) cells [49] were maintained in a 1:1 blend of DMEM and Ham's Nutrient Mixture F12 medium (Invitrogen) plus 10% FBS. All cells were grown in a 5% CO_2_ atmosphere at 37°C in the above media with 100 U/ml penicillin and 100 μg/ml streptomycin.

PHKs and RPE1 cells expressing HPV-16E7, -58E7, -6E7 and -16E7 mutants L22A or C24G or Babe vector were established by retrovirus-mediated infection with the pBabe-puro-based retroviral construct. Experiments were performed with cells within 10 passages.

### RNA extraction, RT-PCR and real-time quantitative PCR (qRT-PCR)

Total cellular RNA was extracted from cells by use of the RNeasy mini kit (Qiagen, Alameda, CA), then reverse-transcribed to cDNA by use of the Improm-II Reverse Transcriptase system kit (Promega, Madison, WI). The PCR primers were as follows:

HPV-16E7, forward, 5′-ATGAGAGGAAACAAC CCAAC-3′

HPV-16E7, reverse, 5′-AGCTAGGGCACACA ATGGTA-3′

β-actin, forward 5′-TGGCATTGCCGACAGGATG CAGAA-3′

β-actin, reverse 5′-CTCGTCATACTCCTGCTTG CTGAT-3′

Quantitative real-time PCR of CIP2A, c-Myc and E2F1, E2F2, E2F3 levels were performed as described [40] using the following oligos:

CIP2A, forward, 5′-GCCACACTGATTCGGTG TTTT-3′

CIP2A, reverse, 5′-TGCCGACAAAGATTTGCC AATA-3′

c-Myc, forward, 5′-GTCAAGAGGCGAACACA CAAC-3′

c-Myc, reverse, 5′-TTGGACGGACAGGATGT ATGC-3′

E2F1, forward, 5′-CATCCCAGGAGGTCACT TCTG-3′

E2F1, reverse, 5′-GACAACAGCGGTTCTT GCTC-3′

E2F2, forward, 5′-ACGGCGCAACCTACAA AGAG-3′

E2F2, reverse, 5′-GTCTGCGTGTAAAGCG AAGT-3′

E2F3, forward, 5′-GGTCCTGGATCTGAACA AGGC-3′

E2F3, reverse, 5′-CCTTCCAGCACGTTG GTGAT-3′

GAPDH, forward, 5′-GCACCGTCAAGGCTGA GAAC-3′

GAPDH, reverse, 5′-TGGTGAAGACGCCAG TGGA-3′

### Western blot assay

Protein samples were analyzed as described [47]. Membranes were incubated with primary antibodies for pRb (554136), p130 (610261), Cdk1 (610038), E2F1 (all BD Biosciences, 554213); CIP2A (novus, NB100–68264); Cdk2 (sc-6248), Cdk4 (sc-260), Cdk6 (sc-177), cyclin A2 (sc-751), cyclin D1 (sc-718), c-Myc (sc-764), E2F2 (sc-633), E2F3 (sc-878), or GAPDH (all Santa Cruz Biotechnology, sc-25778); c-Myc (phosphor S62) (Abcam, ab51156 )

### Cell transfection

RPE1 cells were seeded at 3 × 10^3^ cells in 6-cm dishes and cultured in medium without antibiotics for 24 hr. Chemically modified small interfering RNA (siRNA) oligonucleotides [16] were transfected into cells with use of Lipofectamine RNAimax (Invitrogen). Cells were harvested and used for further experiments at 48 hr after transfection. Plasmid pCMV-E2F1 and the control (Addgene) were transfected into cells with use of Lipofectamine 2000 (Invitrogen).

### Cell proliferation

Cell proliferation assay involved the Cell Counting Kit 8 (Dojindo, Japan) following the manufacturer's recommendation as described [50].

### Flow cytometry

Asynchronous cultures of cells were treated with phosphate buffered saline (PBS) or bleomycin (10 μg/mL) for 24 hr. Cells were harvested, fixed in 70% ethanol, and stained with propidium iodide (PI, 50 μg/mL) in the presence of DNase-free RNase A (70 μg/mL) and analyzed for cell cycle distribution.

For bromodeoxyuridine (BrdU) labeling, RPE1 cells were collected 48 hr after transfection with siRNA of CIP2A or control, then BrdU (final 20 uM) was added to the medium. After an additional 2 hr, cells were harvested and fixed in 70% ethanol. Cells were permeabilized with 2 N HCl-0.5% Triton X-100, neutralized with 0.1 M sodium tetraborate, stained with monoclonal anti-BrdU (BD Biosciences), then with anti-mouse IgG F(ab)2-FITC (Sigma), and counterstained with PBS-PI-RNase A. Immunofluorescent cells were analyzed by use of FACSCalibur (BD) and FCSexpress.

### Statistical analysis

Data are presented as mean ± SD and analyzed by two-tailed Student's *t* test. *P* ≤ 0.05 was considered statistically significant.
